# Electrochromic two-dimensional covalent organic framework with a reversible dark-to-transparent switch

**DOI:** 10.1038/s41467-020-19315-6

**Published:** 2020-11-02

**Authors:** Fei Yu, Wenbo Liu, Si-Wen Ke, Mohamedally Kurmoo, Jing-Lin Zuo, Qichun Zhang

**Affiliations:** 1 School of Materials Science and Engineering, Nanyang Technological University, Singapore, 639798 Singapore; 2 Institute of Advanced Materials and Flexible Electronics (IAMFE), School of Chemistry and Materials Science, Nanjing University of Information Science & Technology, 210044 Nanjing, People’s Republic of China; 3 State Key Laboratory of Coordination Chemistry, School of Chemistry and Chemical Engineering, Collaborative Innovation Center of Advanced Microstructures, Nanjing University, 210093 Nanjing, People’s Republic of China; 4 Institut de Chimie de Strasbourg, CNRS-UMR 7177, Université de Strasbourg, 4 rue Blaise Pascal, 67008 Strasbourg, France; 5 Department of Materials Science and Engineering, City University of Hong Kong, Kowloon, Hong Kong SAR China

**Keywords:** Polymer synthesis, Polymers, Electronic devices

## Abstract

Electrochromic (EC) materials with a dark-to-transmissive switch have great applications in optical communications, infrared wavelength detectors for spacecraft, and infrared camouflage coatings. However, such electroactive materials with high stability and cyclability are rare. Considering the advantages of the donor-acceptor approach (wide-range tuneable band position) and porous two-dimensional (2D) covalent organic framework (COF, well-ordered crystalline framework with stable structure and high surface area), in this work we constructed an extended delocalised π-electron layered dark purple EC-COF-1 by reacting the donor N,N,N′,N′-tetrakis(*p*-aminophenyl)-*p*-benzenediamine (TPBD) with the acceptor 2,1,3-benzothiadiazole-4,7-dicarboxaldehyde (BTDD). A sandwiched device made of EC-COF-1 exhibits the two-band bleaching (370 nm and 574 nm) in the visible region and becomes transparent under the applied potential with an induced absorption centring at 1400 nm. This discovery of a stable dark-to-transmissive switch in COF might open another door for their application in many EC devices for various purposes.

## Abstract (web summary)

Electrochromic materials are important for different optical applications but often these materials show low stability. Here, the authors demonstrate a stable donor-acceptor covalent organic framework which shows a stable dark-to-transmissive switching behaviour.

## Introduction

Recently, organic layered materials with extended π-electron structures, and controllable porosity to mimic graphene and its analogues are becoming the focus of much scientific researches due to the numerous technological attributes—low density, easy preparation, electronic activity, photoactivity associated with the tuneable colours, the low-cost processing and manufacturing, and the friendliness to our environment^1,2^. Generally, the design strategy to construct electronic and optical materials is to create covalent bonds between two redox-active building blocks^3^. If both electron donating (D) and electron accepting (A) building units are simultaneously used in one structure, a D–A type-conjugated covalent organic framework (COF) is formed^4–7^. By selecting the suitable D and A units with reasonable energy levels (redox potentials), the COF with the desired electrical, optical, or combined optoelectronic properties could be achieved^8–10^. Recent reports have demonstrated the applicability of functional D–A COF as active elements in organic photovoltaics (OPV)^11,12^, photoconductive devices^13^, photocatalysis^14–20^, and two-photon-induced florescence^21^. Beside benefitting from their ordered micro- or meso-pores^22–24^, COFs are also used as external-stimuli-responsive materials to sense light^25^, solvent^26,27^, and pH^28–31^. Although the energy levels of D–A polymers can be tuned to reach the desired valence and/or conduction band positions for the purpose to display desired EC properties^32–35^, the application of D-A COF in electrochromic devices (ECD) is rare^36,37^. However, for most EC products, such as eyewear, smart windows, and display devices, neutral-state black devices integrated with bleached states are highly desirable^38^. Nevertheless, to the best of our knowledge, black colour currently remains a big challenge due to the special demand for broadband absorption across the whole visible region. Such gap strongly encourages us to develop COF with the ordered pores and the extended regular π–π stacking structures for potential application in electrochemistry and photoelectrochemistry fields.

**Fig. 1:**
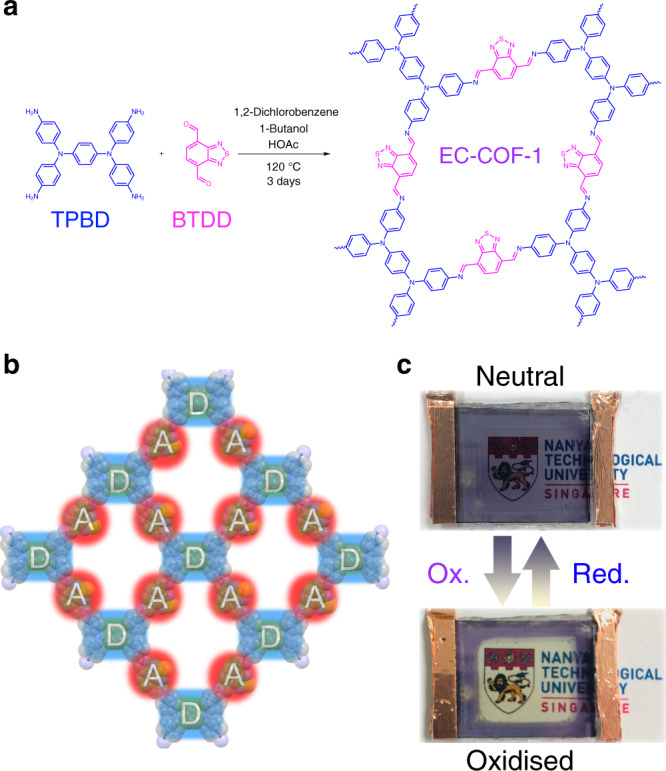
Synthesis and device of EC-COF-1. **a** Synthetic route of EC-COF-1. **b** Schematic representation of a layer structure of donor (D)–acceptor (A) EC-COF-1. **c** Colour switching of the EC-COF-1 EC device.

Here, we show a D-A two-dimensional (2D) COF with N,N,N′,N′-tetrakis(*p*-aminophenyl)-*p*-benzenediamine (TPBD)^39^ as donor, and 2,1,3-benzothiadiazole-4,7-dicarboxaldehyde (BTDD) as acceptor^40^, was prepared (Fig. 1 and Supplementary Fig. 1) and has been employed as an active element for EC device (Fig. 1c). When the voltage is ramped from −1.8 to +2 V, the colour of the as-fabricated 2D COF films accordingly change from opaque black to transparent. Note that under our experimental condition, all EC devices are stable and can be reversibly switched. The typical modulation of the electronic structure of the COF has been provided in Fig. 1c.

## Results

### COF design and synthesis

To obtain efficient electrical response, COF materials should not only have sufficient oscillator strength during charge transfer transitions, but also be sensitive to the changes of polarity in the pores. The COF with electron-rich donors and electron-deficient acceptors as building blocks might be ideal candidates for such applications, because they would provide us more opportunities to tune their band gap and electrochemical activity. Since our previous results have already demonstrated that triphenylamine units could easily realise well-ordered frameworks with large crystal domains^25^, the same strategy will be extended in the present research. Besides, this type of units might enable us to optimise the EC response within a single COF family. Moreover, we believe that the tuneable electron deficiency in aldehyde counterparts would produce the electronic transitions, with the varying degrees of charge transfer across the conjugated imine bond and decrease the energy gap to form the dark-coloured COF (Supplementary Fig. 2).

The building units of TPBD and BTDD were synthesised according to the reported methods with slight modifications (see in Supporting Information)^39,40^. The 2D COF was prepared as follows: after dissolving TPBD and BTDD in a mixed solvent of 1,2-dichlorobenzene:*n*-butanol (v:v = 1:1) and ultrasonicating 20 min to generate a dark purple solution, acetic acid (6 M) was added, followed by rapid freezing to 77 K, degassed through three cycles of freeze–pump–thaw, and sealed under vacuum. After the sealed tube was heated at 120 °C for 72 h, the targeted EC-COF-1 was formed as a dark purple precipitate.

EC-COF-1 films were deposited on transparent conducting indium tin oxide (ITO) electrodes by direct liquid/solid interface solvothermal method at 120 °C. ITO electrodes were cut to the appropriate size (1.0 × 1.5 cm) and placed vertically in a glass tube. The as-fabricated EC-COF-1 film on the ITO electrode displays blue-purple colour with the well adhesion ability.

### Structure characterisation

**Fig. 2:**
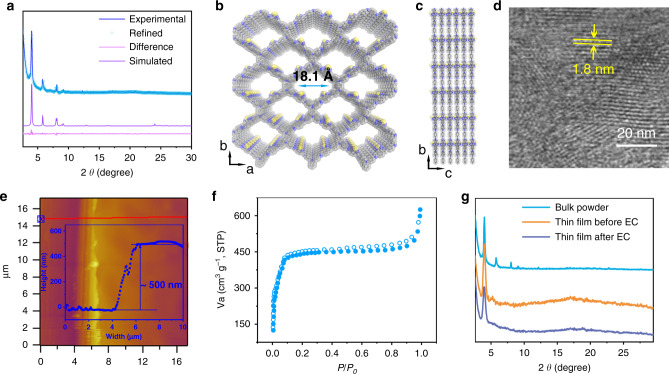
Structural aspects of EC-COF-1. **a** Observed (purple line), Pawley refinement (red cross), difference (blue line), and eclipsed AA simulation (green line) PXRD patterns. Simulated structure of EC-COF-1: **b** top and **c** side views. (Colours: blue—N, grey—C, yellow—S). **d** HR-TEM image. **e** AFM image with ~500 nm height. Porous properties: **f** N_2_ sorption isotherm (solid iris blue ball for adsorption, and hollow iris blue ball for desorption). **g** The X-ray diffraction pattern of the as-synthesised bulk powder (black line), thin film before EC (red line) and thin film after EC (blue line) of EC-COF-1.

The powder X-ray diffraction (PXRD) pattern of the as-synthesised EC-COF-1 (Fig. 2a) shows several distinct Bragg reflections with 2*θ* approximately at 4.0, 5.8, 8.1, and 9.0°, consistent with that simulated for a model structure. Furthermore, Pawley refinement was used to extract *C*2 space group, and a unit cell of *a* = 31.96, *b* = 30.87, *c* = 4.72 Å, and *β* = 92.3°. The good match between the experimental result and the simulated pattern is further confirmed as judged by their difference (Fig. 2a–c and Supplementary Table 1). The suitable sample for transmission electron microscopy (TEM) was prepared through immersing ITO electrode into EtOH, where the films automatically detached from the ITO substrates. The TEM image (Fig. 2d) of the film clearly displays many parallel lines with the neighbouring distance of ~1.8 nm, attributing to a dominant *hkl* of an ordered structure of the EC-COF-1. The homogenous topography of the as-fabricated film is investigated through atomic force microscope (AFM; Fig. 2e and Supplementary Fig. 3), where the thickness of the EC-COF-1 film lies in the range 500 ± 20 nm. This result further provides solid experimental evidence of the high crystalline and uniformity of the as-fabricated COF films. Attenuated total reflection Fourier transform infrared spectra revealed the absence of both ν(N − H) from TPBD in the range of 3450–3010 cm^−1^ and ν(C = O) at 1670 cm^−1^ from BTDD, while the emerging band at 1620 cm^−1^ is assigned to ν(C = N) for EC-COF-1 (Supplementary Fig. 4). The Brunauer−Emmett−Teller measurement showed a non-hysteretic sorption isotherm for N_2_ gas at 77 K with a surface area of 915 m^2^ g^−1^ and a pore volume of 0.64 cm^3^ g^−1^ (Fig. 2f and Supplementary Fig. 5). The non-local density function theory^31–42^ was employed to estimate a pore size distribution of ~1.8 nm (Supplementary Fig. 5). The X-ray diffraction (XRD) patterns of the as-prepared films before and after electrochemical oxidation (Fig. 2g) clearly indicated that there were almost no changes, suggesting that the structure of the as-prepared EC-COF-1 film has high stability. Thermogravimetric analysis revealed that EC-COF-1 has high thermal stability up to 405 °C (Supplementary Fig. 6).

### Band structure calculation

**Fig. 3:**
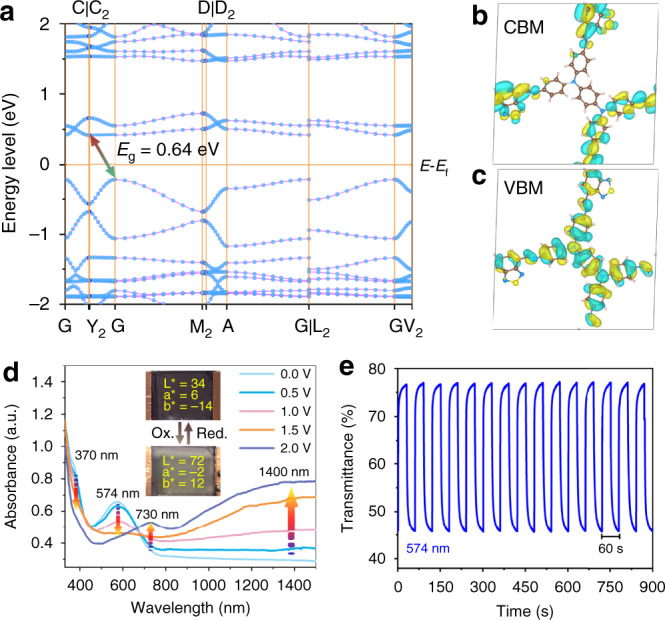
Electronic structure and characteristics of EC-COF-1. **a** Band structures. The coordinates of high-symmetry points are Γ—C|C_2_—Y_2_—Γ—M_2_—D|D_2_—A—Γ|L_2_—Γ—V_2_ (orange lines). **b** HOMO at VB. **c** LUMO at CB. **d** Changes in the optical spectrum of EC-COF-1 film as a function of applied potential. **e** Repetitive display of transmittance at 574 nm (blue line) for the voltage between –1.8 and +2.0 V.

The electronic energy diagram of the band structure calculated by the PBE-D3 process clearly suggests that pristine EC-COF-1 is a semiconductor with an apparent indirect energy band gap of 0.64 eV (Fig. 3a and Supplementary Fig. 7)^43^, which is consistent with the observed gap of 0.75 eV from solid-state ultraviolet–visible–near-infrared (UV–vis–NIR) spectrum (Supplementary Fig. 8). The bandwidths of the valence and the conduction bands are 0.34 and 0.15 eV, respectively. The HOMO of the valence band maximum (VBM) is predominantly located on the TPBD donor node, while the LUMO of the conduction band minimum (CBM) is delocalised over the BTDD acceptor unit, confirming the π-conjugated feature (Fig. 3b, c). Furthermore, the CBM and VBM dominantly constitute of the atomic orbitals of C and N, which supports the creation of expanded π-conjugation, since these energy levels were observed through the partial density of states (Supplementary Figs. 9 and 10)^44–46^.

### Electrochromism

A quasi-solid-state device was constructed to evaluate the potential application of the EC-COF-1 film as an EC electrode. The EC device has a sandwiched configuration (Figs. 1b and 3d), where an EC-COF-1-covered ITO plate was employed as the working electrode, another blank ITO glass as the counter electrode, and a LiClO_4_/propylene carbonate-based PMMA polymer gel as a quasi-solid electrolyte, which was injected into the space between two electrodes. When an applied external potential across the device is gradually increased, both absorption bands at 370 and 574 nm proportionally decrease. Meanwhile, the concomitant formation of new absorption arose in the NIR region (Fig. 3d), indicating the formation of radical cations (polarons) and further evolving into dications (bipolarons) at higher bias voltages (Supplementary Fig. 11). These changes can be clearly observed in the transmittance (Δ*T*%) of 33% at 574 nm and 12% at 730 nm. Moreover, the stability of EC devices was also studied, where Δ*T*% dropped only ca. 0.3% at 574 nm and 0.5% at 730 nm after 15 cycles of switching potential. Also, good stability in 200 cycles was observed under cyclic voltammetry test. Moreover, the XRD pattern at the hkl (100) Bragg reflection was retained, indicating that the EC-COF-1 structure is stable after EC performances (Figs. 2g and 3e, and Supplementary Figs. 12 and 13). The response time is obtained from the Δ*T*% experiments when the contrast ratio reach over 90% of its maximum of EC-COF-1 ECD between bleached and coloured states. Colouring (*t*_c_) and bleaching (*t*_b_) times of film electrodes at 574 and 730 nm were 1.8/7.2 and 2.6/3.5 s, respectively (Supplementary Figs. 14 and 15). The colouration efficiencies at 574 and 730 nm were 284 and 246 cm^2^ C^−1^, respectively. Since NIR switching has some important applications in several fields, such as infrared wavelength detectors for spacecraft, optical communications, and biomedicals^47–51^, our D–A COF with the distinct advantage to significantly reduce the energy band gap (Fig. 3a) could be treated as a promising visible-to-NIR EC material.

The relative luminance changes during potential programming were studied through measuring the brightness of the transmitted light as a percentage of that of a light source calculated against the eye sensitivity. In the process of electrochemical oxidation, the colour of EC-COF-1 films switched from an opaque blue-purple state to strongly transparent appearance with a relative luminance change of up to 36.5% (Supplementary Fig. 16). A detailed colorimetry study of the blue-purple colour of the neutral-state EC-COF-1 suggests significantly negative b* value and in particular small a* value compared to b*, where L*a*b* values of the neutral and oxidised states can be figured out from the CIE 1976 L*a*b* colour model. By oxidation, the L* value jumps from 34 to 72, while the a* and b* values change from 6 to −2 and −14 to 12, respectively (Fig. 3d and Supplementary Fig. 16). These results imply that EC-COF-1 transforms into a transmissive state, where the human eye can hardly perceive the residual colour, suggesting that the EC device reaches nearly the ‘white point’.

Different from most EC materials with colour changes from light (neutral state) to dark (oxidised state), the D–A approach could allow us to construct EC COF materials with two-band absorption (deep absorption, i.e., blue-purple) in the visible region (neutral state), which becomes transparent after an electric field is applied, and simultaneously, NIR absorption arises. This approach allows us to ‘merge’ these bands together for the synthesis of highly saturated dark-coloured materials. Furthermore, theoretical work suggests that the ground state of  the oxidised D–A system contains the local energy levels within the band gap, which could rationalise the presence of the low energy NIR bands in the optical spectrum.

## Discussion

We have demonstrated a successful D–A approach in constructing COF with a reversible electrochromic property switching from blue-purple to transparent. We believe that their D–A structure, stable crystallinity, high surface area, reversible transparency in the visible region, and the opacity in the NIR region are key factors to allow us to obtain such dark-to-transmissive switch. Together with other appealing advantages of COF such as the ease of self-assembly and low weight, we believe that such materials would have great application in diverse EC devices and stimuli-responsive instruments.

## Methods

### The synthesis of EC-COF-1 bulk powder

The synthesis of COF bulk powder was performed under vacuum in polytetrafluoroethylene-sealed glass reaction tubes (20 mL). Solvents and acetic acid were obtained in highly pure grades from commercial suppliers and were, unless shipped under argon, degassed, and saturated with argon prior to use. TPBD (23.2 mg, 50 μmol) and BTDD (19.3 mg, 100 μmol) were filled into a reaction tube, followed by the addition of a mixed solvent of 1,2-dichlorobenzene:*n*-butanol (v:v = 1:1 mL). After ultrasonicating 20 min, a dark purple solution was formed, followed by the addition of acetic acid (200 µL, 6 M), rapid freezing to 77 K, degassed through three freeze–pump–thaw cycles for three times, and sealed under vacuum. Then, the sealed tube was heated at 120 °C for 72 h. After cooling to room temperature, the precipitate was collected by filtration, washed with MeCN, and dried in air to yield a dark purple precipitate.

### EC-COF-1 thin film synthesis

COF thin films were prepared in a 20 mL glass reaction tube of 12 mm diameter. ITO-coated glass (10–12 Ω/ϒ) substrates were cleaned with detergent solution, water, acetone, and isopropanol, and then, activated with an O_2_-plasma for 10 min directly before use. The substrates were placed vertically in a glass tube. TPBD (4.6 mg, 10 μmol) and BTDD (3.8 mg, 20 μmol) were filled into the tube, followed by the addition of a mixed solvent of 1,2-dichlorobenzene:*n*-butanol (v:v = 2:2 mL), and ultrasonicating 20 min to generate a dark purple solution. An ITO substrate was inserted and acetic acid (200 µL, 6 M) was added, followed by rapid freezing to 77 K, degassed through freeze–pump–thaw cycle for three times. The tube was sealed and heated to 120 °C for 72 h. After cooling to room temperature, the substrate was immersed in dry MeCN and dried under N_2_. Thinner films were grown using shorter reaction times ranging from 4 h to 2 days.

### Structure characterisation

PXRD patterns were conducted on a PANalytical X’Pert Pro MPD diffractometer using Cu K*α* radiation (*λ* = 1.5406 Å) and operating at 40 kV and 40 mA between 2 and 30° (2*θ*). Thin film XRD patterns were conducted on Bruker D8 Discover with Ni-filtered Cu K*α* radiation (*λ* = 1.5406 Å) and a LynxEye position-sensitive detector. TEM was performed with a JEM-2100 (JEOL Ltd., Japan) with an accelerating voltage of 200 kV.

### Optical absorption spectroscopy

UV–vis–NIR spectra were recorded on a Perkin-Elmer Lambda 950 spectrometer equipped with a 150 mm InGaAs integrating sphere. Time-resolved absorption measurements were performed at fixed detector gain and slit settings. Diffuse reflectance spectra were collected with a Praying Mantis (Harrick) accessory and were referenced to barium sulphate powder as white standard. The specular reflection of the sample surface was removed from the signal by spatial filtering.

## Supplementary information

Supplementary Information

## Data Availability

The data that support the findings of this study are available within the article and Supplementary Information files, or available from the corresponding authors on request. Source data are provided with this paper.

## Source data

Source Data
