# Supplementary material for: Electrochromic two-dimensional covalent organic framework with a reversible dark-to-transparent switch
Source: Nat Commun. 2020 Nov 2;11:5534. doi: 10.1038/s41467-020-19315-6 (PMC7608553; doi:10.1038/s41467-020-19315-6)
Supplement: Supplementary file 1 — Supplementary Information [file 41467_2020_19315_MOESM1_ESM.pdf]

Supplementary Information

Electrochromic Two-Dimensional Covalent Organic Framework with a Reversible Dark-to-Transparent Switch

Fei Yu[1,2], Wenbo Liu[1], Si-Wen Ke[3], Mohamedally Kurmoo[4], Jing-Lin Zuo[3]* and Qichun Zhang[1,5]*

[1]School of Materials Science and Engineering, Nanyang Technological University, Singapore 639798, Singapore. [2]Institute of Advanced Materials and Flexible Electronics (IAMFE), School of Chemistry and Materials Science, Nanjing University of Information Science & Technology, Nanjing, 210044, P. R. China. [3]State Key Laboratory of Coordination Chemistry, School of Chemistry and Chemical Engineering, Collaborative Innovation Center of Advanced Microstructures, Nanjing University, Nanjing 210093, P. R. China. [4]Institut de Chimie de Strasbourg, CNRS-UMR 7177, Université de Strasbourg, 4 rue Blaise Pascal, 67008 Strasbourg, France. [5]Department of Materials Science and Engineering, City University of Hong Kong Kowloon, Hong Kong SAR, China.

*E-mail: zuojl@nju.edu.cn; qcizhang@cityu.edu.hk

Contents

Supplementary methods

Chemicals, analytical techniques and instruments, and preparation of BTDD and TPBD.

Supplementary Table

Supplementary Table 1. Fractional atomic coordinates of structural model of **EC-COF-1**.

Supplementary Figures

Supplementary Fig. 1. Schematic representation of a layer structure of Donor (D)−Acceptor (A) **EC-COF-1**.

Supplementary Fig. 2. DFT calculations.

Supplementary Fig. 3. Atomic Force Microscope images of **EC-COF-1** film.

Supplementary Fig. 4. ATR FTIR spectra of **EC-COF-1** (black), TABD (red) and BTDD (blue).

Supplementary Fig. 5. Pore size distribution of **EC-COF-1**.

Supplementary Fig. 6. Thermogravimetric analysis (TGA) of **EC-COF-1**.

Supplementary Fig. 7. The Brillouin zone path of **EC-COF-1**.

Supplementary Fig. 8. The Tauc plot of BTDD (yellow), TPBD (blue) and **EC-COF-1** (purple).

Supplementary Fig. 9. The total charge density 2D slice of **EC-COF-1**.

Supplementary Fig. 10. The TDOS of **EC-COF-1** and pDOS projected onto each constituent element.

Supplementary Fig. 11. Illustration of **a** electronic, **b** absorption and electronic transition changes of **EC-COF-1** under oxidative doping.

Supplementary Fig. 12. Repetitive display of induced current and transmittance for the voltage between –1.8 and +2.0 V at 730 nm.

Supplementary Fig. 13. Cyclic voltammogram scans of the **EC-COF-1** film.

Supplementary Fig. 14. Optical transmittance changes in films monitored at 574 nm.

Supplementary Fig. 15. Optical transmittance changes in films monitored at 730 nm.

Supplementary Fig. 16. Relative Luminance (%) as a function of applied potential for **EC-COF-1**.

Supplementary References

Supplementary methods

Chemicals. All starting materials and solvents, unless specified, were obtained from Sigma-Aldrich Chemicals and used without further purification.

Analytical techniques and instruments. Attenuated total reflectance Fourier-transform infrared spectroscopy (ATR FTIR) of solid samples were performed on a PerkinElmer Frontier spectrometer. Nuclear magnetic resonance (NMR) spectra were recorded with a Bruker AV 300 Spectrometer at 300 MHz (^{1}H NMR). Powder X-ray diffraction (PXRD) patterns were conducted on PANalytical X'Pert Pro MPD diffractometer using Cu Kα radiation (λ = 1.5406 Å), and operating at 40 kV and 40 mA between 2 and 30° (2θ). Thin film X-ray diffraction (XRD) patterns were conducted on Bruker D8 Discover with Ni-filtered Cu Kα radiation (λ = 1.5406 Å) and a LynxEye position-sensitive detector. Transmission electron microscope (TEM) was conducted on a JEM-2100 (JEOL Ltd., Japan) with an accelerating voltage of 200 kV. Thermogravimetric analyses (TGA) were carried out on an SDT Q600 TG-DTA analyzer under N_2 atmosphere at a heating rate of 10 ºC min^{-1} within a temperature range of 40-800 °C. Low-pressure volumetric N_2 gas adsorption measurements were performed on a Quantachrome Quadrasorb automatic volumetric instrument. Before measurement, all samples were degassed in vacuum at room temperature for 24 h. The Brunauer-Emmett-Teller (BET) method was utilised to calculate the specific surface areas. By using the non-local density functional theory (NLDFT) model, the pore size distribution was derived from the sorption curve.

Synthetic procedures

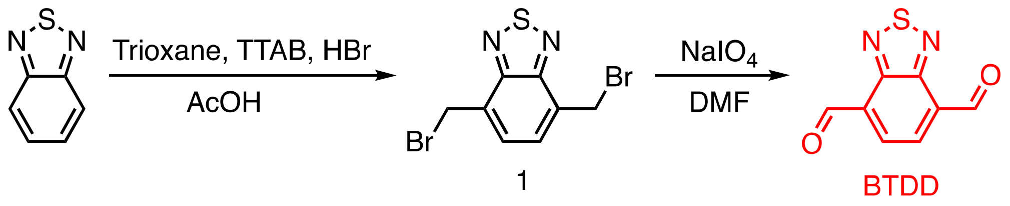

Scheme S1. Synthetic route to BTDD.

Synthesis of 4,7-Bis(bromomethyl)benzo[c][1,2,5]thiadiazole (1)[1]. To the stirred solution of 2,1,3-benzothiadiazole (4.0 g, 29.4 mmol) in 48% aqueous hydrobromic acid

(HBr, 80 mL) and glacial acetic acid (20 mL), trioxane (3.2 g, 146.0 mmol) and trimethyl(tetradecyl)-ammonium bromide (0.6 g) were added at room temperature. Then, this solution was refluxed for 36 h. After cooling, the solid was filtered and washed with water and ethanol, and then dried under vacuum to afford compound **1** as a white solid. Yield: (6.5 g, 68%). ^{1}H NMR (300 MHz, $CDCl_3$) δ 7.63 (s, 2 H), 4.97 (d, 4 H).

Synthesis of Benzo[c][1,2,5]thiadiazole-4,7-dicarbaldehyde (BTDD)[1]. Compound **1** (6.0 g, 18.6 mmol) and sodium periodate ($NaIO_4$) (2.0 g, 9.7 mmol) were placed in a round bottom flask under argon atmosphere. Then, 60 mL of N,N-dimethylformamide (DMF) was added. After all compounds dissolved, the as-resulted solution was slowly heated up to 150 °C under stirring. The progress of the reaction was monitored by thin layer chromatography (TLC) through the comparison with the starting material (hexane:ethyl acetate (EA), 9:1 v/v). Within 90 min, the starting material was completely disappeared and two new spots were found in TLC. Then, the reaction was stopped and the solvent was removed by rotary evaporation. The as-obtained solid mixture was poured into 100 mL water. The resulted mixture was stirred for 40 min at RT, and then was extracted with EA for three times (50 mL × 3). The combined organic layer was washed with brine and dried over anhydrous Na_2SO_4. After filtration and evaporation, the crude product was purified by column chromatography (silica gel, Hexane:EA, 9:1 v/v) to afford pure compound BTDD as an orange solid. Yield: (1.2 g, 33%). ^{1}H NMR (300 MHz, $CDCl_3$) δ 10.90 (s, 2 H), 8.39 (s, 2 H).

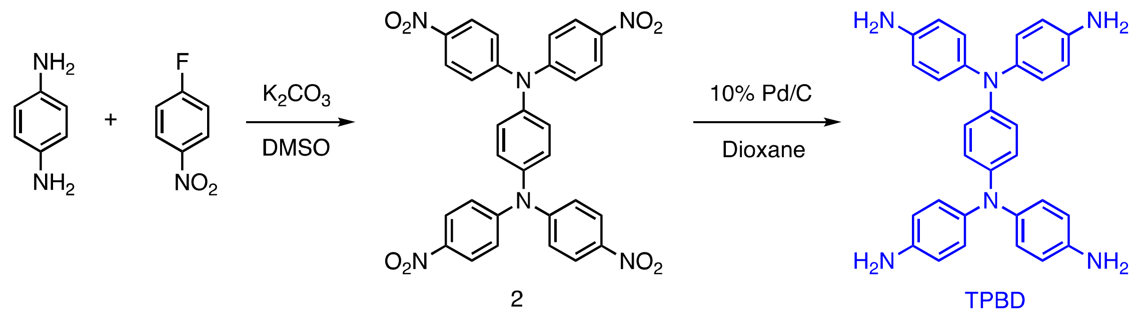

Scheme S2. Synthetic route to TPBD.

TPBD[2] was synthesised as follows: compound **2** (N,N,N',N'-tetrakis(*p*-nitrophenyl)-*p*-phenylenediamine) was prepared by reacting *p*-phenylenediamine with 4-

fluoronitrobenzene in dimethyl sulphoxide (DMSO) through heating under the existence of K_2CO_3 and N_2 atmosphere. Compound N,N,N,N'-tetrakis(*p*-aminophenyl)-*p*-phenylenediamine (TPBD) was prepared by catalytic hydrogenation of **2** in dioxane. The synthetic route is described in Scheme 2.

Structure modelling: The structure of **EC-COF-1** was determined by modelling, powder indexing and Pawley refinement based on powder X-ray diffraction (PXRD) pattern. The resulting structure was geometrically optimised using the Forcite module, with a Universal forcefield. The as-obtained model was in good agreement with experimental data. Pawley refinement was performed on the eclipsed (AA) COFs model in space group *C* 2 against experimental data. The refinement was based on the Debye-Scherrer geometry and the Thompson-Cox-Hastings peak profile function. The refinement results are depicted in Figure 3a, and the final atomic coordination is enumerated in Table S1.

Band structure calculations: All of the electronic structure calculations conducted in this research, including optimization of lattice parameters and atomic coordinates, were performed within the framework of density functional theory (DFT) as implemented in the Vienna ab initio simulation package (VASP 5.3.5)[3] using the projector augmented wave (PAW)[4] method and the Perdew−Burke−Ernzerhof (PBE)[5] exchange-correlation functional. London dispersion correction was further applied with Grimme's D3 approach[6], and the Heyd−Scuseria− Ernzerhof (HSE06)[7] hybrid functional was used to obtain accurate band gaps. The cut-off energy of 500 eV for the plane-wave basis set was used in lattice and atomic coordinate optimizations, and 600 eV was used in static calculations. The convergence criterion for forces on atoms during optimizations was set to 0.01 eV $Å^{-1}$, while the energy convergence criterion in the self-consistent field iteration was set to 10^{-5} eV for optimizations and 10^{-6} eV for static calculations. The *k*-meshes of 1 × 1 × 3 were used in the optimization of pristine **EC-COF-1**.

Colouration efficiency. The maximum absorption wavelength (λ_{max}) is determined from the maximum absorption band in the absorption spectroscopy of an electrochromic material either in solution or solid phase. The maximum colour contrast or transmittance change

($\Delta\%T$, %) refers to the maximum optical contrast, determined by the difference between the highest and the lowest transmittance at the target colour, normally at λ_{max}

$$\Delta\%T = T_b - T_c$$

where T_b and T_c are the transmittance (%) at bleached and coloured state, respectively. The optical density (ΔOD) is determined at a specific wavelength, in general at λ_{max}, by using $\%T$ values for the electrochromic layer at the reduced or the coloured state, using

$$\Delta \mathrm{OD} = \log(T_b/T_c)$$

The electrochromic colouration efficiency (CE) is the change in ΔOD at a given wavelength (λ) per injected (reduction) or ejected (oxidation) charge (Q_d) as represented by the following[8-9]

$$\mathrm{CE}(\lambda) = \Delta \mathrm{OD}(\lambda)/(Q/A) = \log(T_b/T_c)_\lambda/(Q/A)$$

where ΔOD(λ) is the change in the optical density at a given λ, Q is the injected/ ejected charge, and A is the electrode area[6]. To evaluate the OM of electrochromism, the transmittance change of electrochromic materials can be determined over time at V_{OFF}. Often it is determined as the time to maintain the transmittance (or contrast) up to 90% of the original state. Otherwise it is also determined as the refreshing time to keep up the original transmittance.

Supplementary Table

Supplementary Table 1. Fractional atomic coordinates of structural model of **EC-COF-1** with eclipsed (AA) stacking mode, resulting from Pawley refinement against experimental PXRD data.

**EC-COF-1**			
Monoclinic, *C* 2, a = 31.9559 Å, b = 30.8745 Å, c = 4.7232 Å, $\alpha = \gamma$ = 90.00°, β = 92.30°.			
Atom	*x*	*y*	*z*
C1	0.53132	-0.10155	0.65062
C2	0.53132	-0.05649	0.65039
C3	0.53834	-0.19372	0.48513
C4	0.42867	-0.17716	0.67113
C5	0.39105	-0.19944	0.68279
C6	0.38454	-0.23919	0.54111
C7	0.41781	-0.25562	0.38437
C8	0.45533	-0.23335	0.37066
C9	0.4616	0.03537	0.52457
C10	0.57029	0.01753	0.31449
C11	0.60842	0.03848	0.29437
C12	0.61676	0.078	0.43477
C13	0.58445	0.09621	0.592
C14	0.54618	0.07531	0.61264
C15	0.67189	0.12594	0.55816
C16	0.71345	0.14375	0.52169
C17	0.74526	0.12393	0.35839
C18	0.78576	0.14473	0.33754
C19	0.79496	0.18532	0.47308
C20	0.76346	0.20307	0.63264
C21	0.72448	0.18262	0.65754

C22	0.66438	-0.29454	0.55746
C23	0.5	-0.12491	0.5
C24	0.5	-0.03311	0.5
N1	0.65655	0.09529	0.40014
N2	0.65456	-0.25862	0.43036
N3	0.1871	0.12174	0.8154
N4	0.25704	0.08554	0.77909
N5	0.5	-0.17105	0.5
N6	0.5	0.0131	0.5
S1	0.2121	0.07845	0.91606
H1	0.5559	-0.11893	0.76984
H2	0.55597	-0.03908	0.76873
H3	0.4328	-0.14657	0.78361
H4	0.36562	-0.18681	0.8061
H5	0.41423	-0.28587	0.26676
H6	0.48024	-0.24642	0.24471
H7	0.56481	-0.01313	0.20535
H8	0.63306	0.02486	0.16824
H9	0.58929	0.1269	0.7029
H10	0.52202	0.08971	0.73914
H11	0.65486	0.14066	0.73357
H12	0.26989	-0.26631	0.74103
H13	0.70123	0.19799	0.78816
H14	0.64323	-0.31213	0.69552

Supplementary Figures

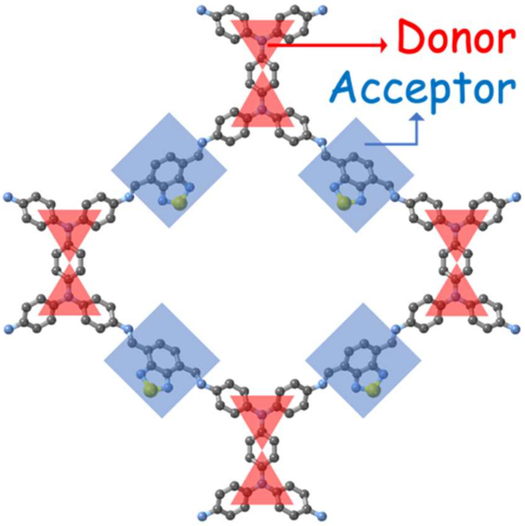

Supplementary Fig. 1. Schematic representation of a layer structure of Donor (D)−Acceptor (A) **EC-COF-1**.

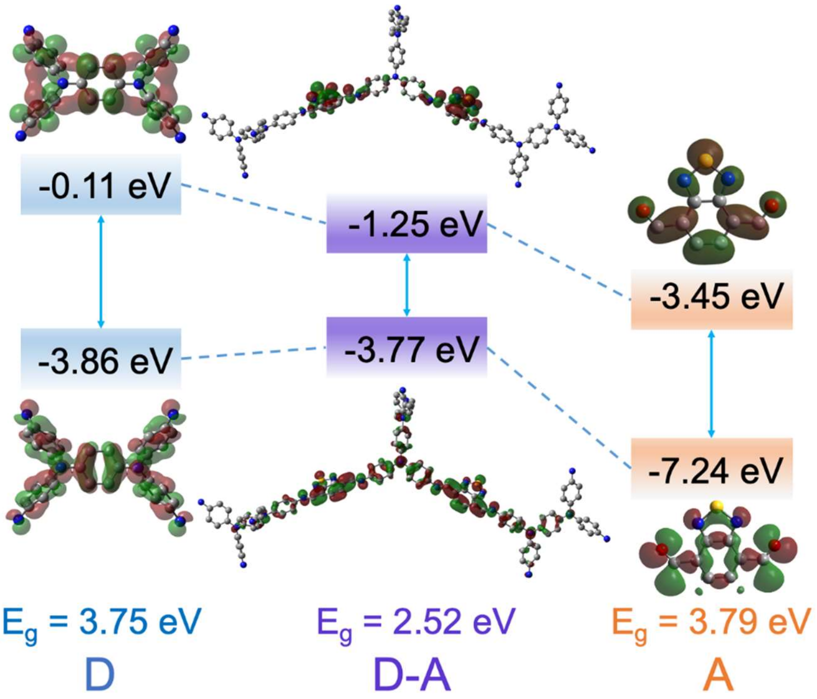

Supplementary Fig. 2. DFT calculations. The calculations were performed on b3lyp/6-31g**. The energy gap of **EC-COF-1** (D-A), TPBD (D) and BTDD (A) are 2.52, 3.75 and 3.79 eV, respectively.

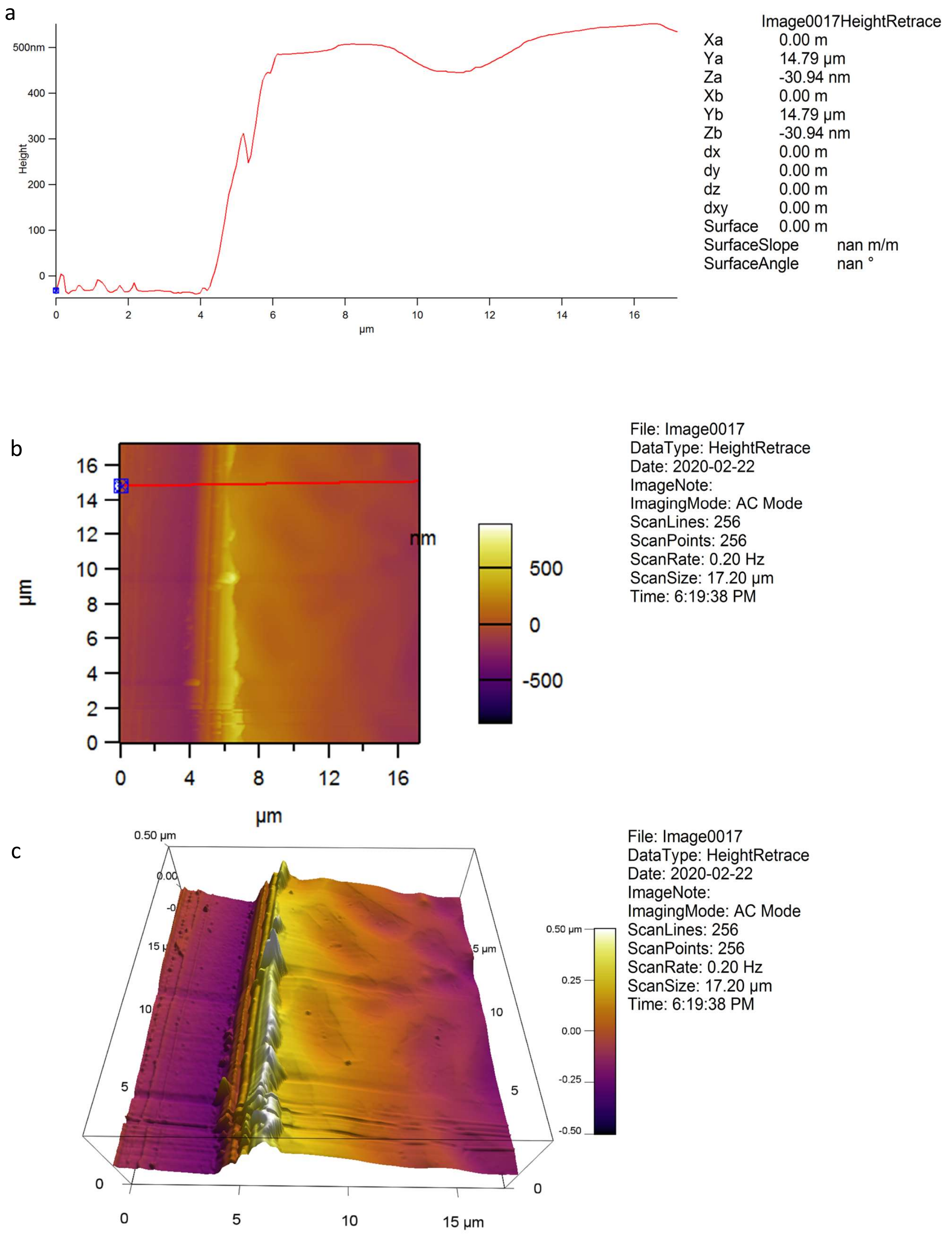

Supplementary Fig. 3. Atomic Force Microscope images of **EC-COF-1** film. **a** The height of **EC-COF-1** film. **b** 2D image of **EC-COF-1** film. **c** 3D image of **EC-COF-1** film.

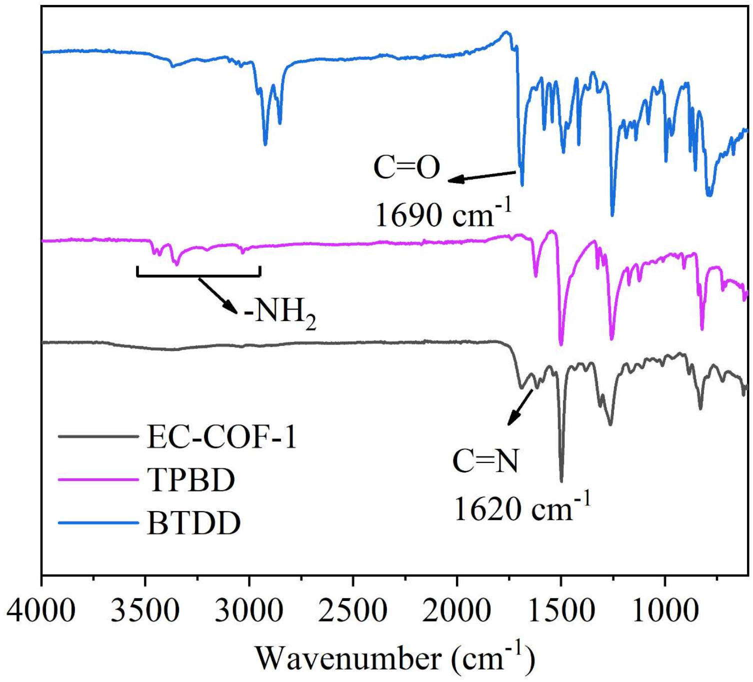

Supplementary Fig. 4. Full width stacked ATR FTIR spectra of **EC-COF-1** (black), TPBD (red) and BTDD (blue).

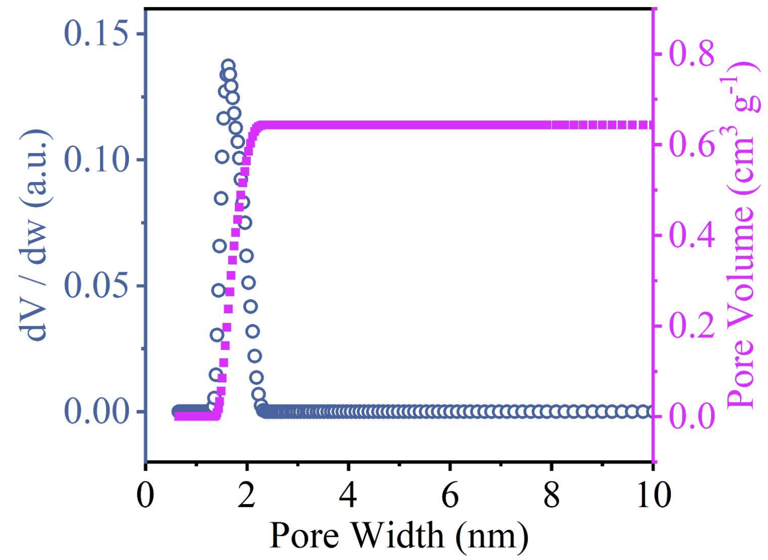

Supplementary Fig. 5. Pore size distribution of **EC-COF-1**.

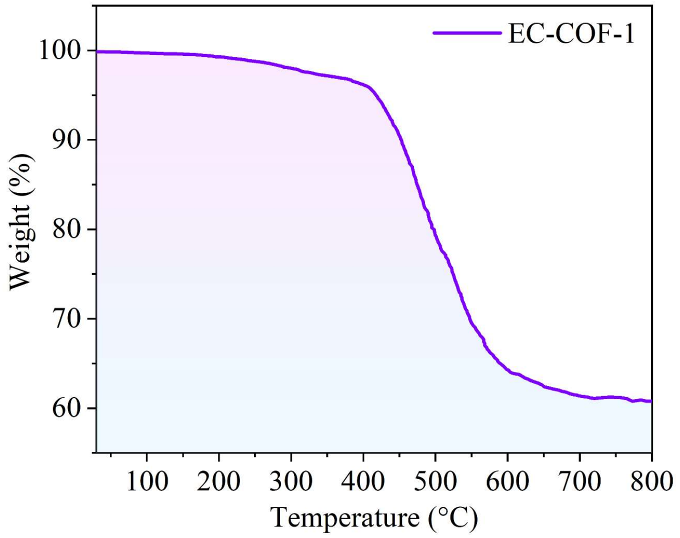

Supplementary Fig. 6. Thermogravimetric analysis (TGA) showing that the decomposition of **EC-COF-1** starts at a temperature of 405 °C.

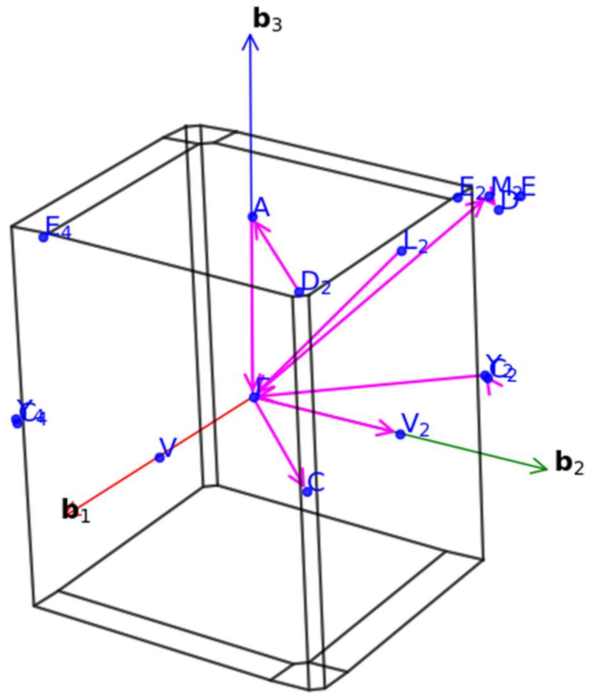

Supplementary Fig. 7. The Brillouin zone path of **EC-COF-1**.

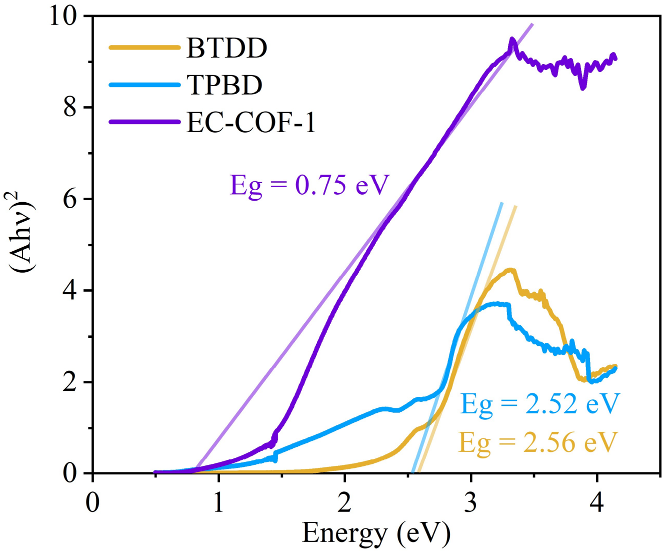

Supplementary Fig. 8. The Tauc plot of BTDD (yellow), TPBD (blue) and **EC-COF-1** (purple). The energy gap for BTDD, TPBD and **EC-COF-1** are 2.56, 2.52 and 0.75 eV, respectively. The energy gap of the **EC-COF-1** is consisted with the band structure calculations.

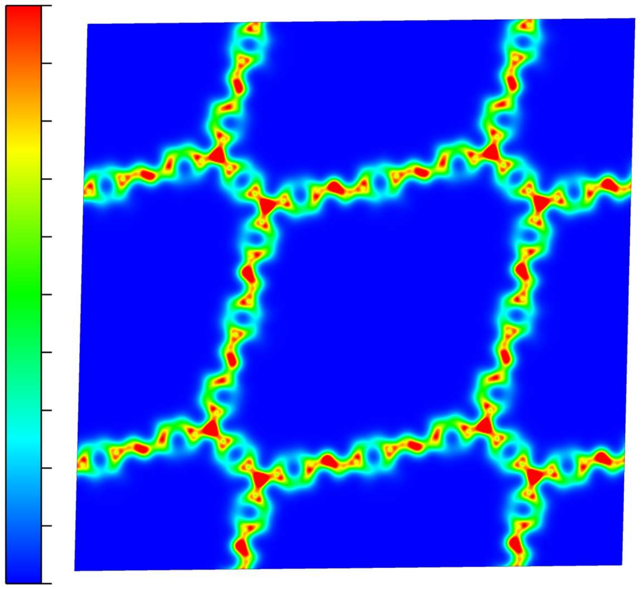

Supplementary Fig. 9. The total charge density 2D slice of **EC-COF-1**. The Miller indices is (0 0 1) and the saturation levels (max: 0.3097, min: -0.0292).

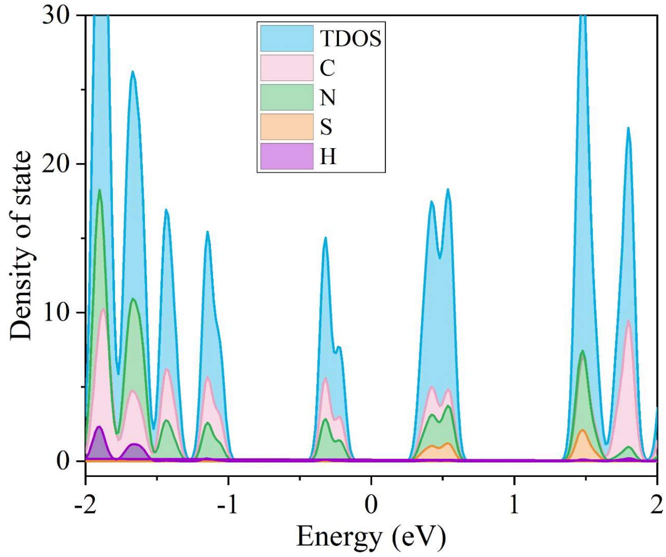

Supplementary Fig. 10. The TDOS of **EC-COF-1** and pDOS projected onto each constituent element.

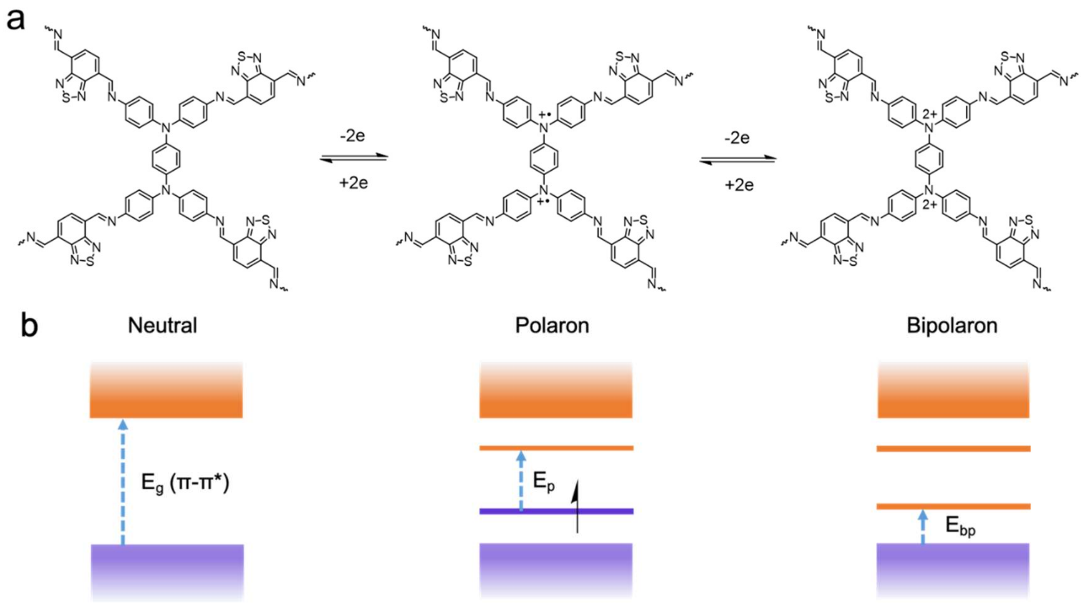

Supplementary Fig. 11. Illustration of **a** electronic, **b** absorption and electronic transition changes of **EC-COF-1** under oxidative doping.

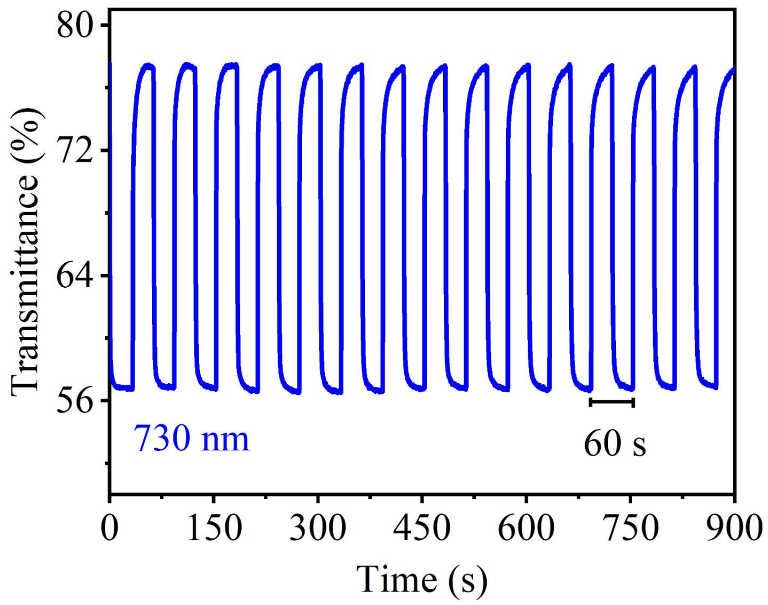

Supplementary Fig. 12. Repetitive display of induced current and transmittance for the voltage between -1.8 and 2.0 V at 730 nm.

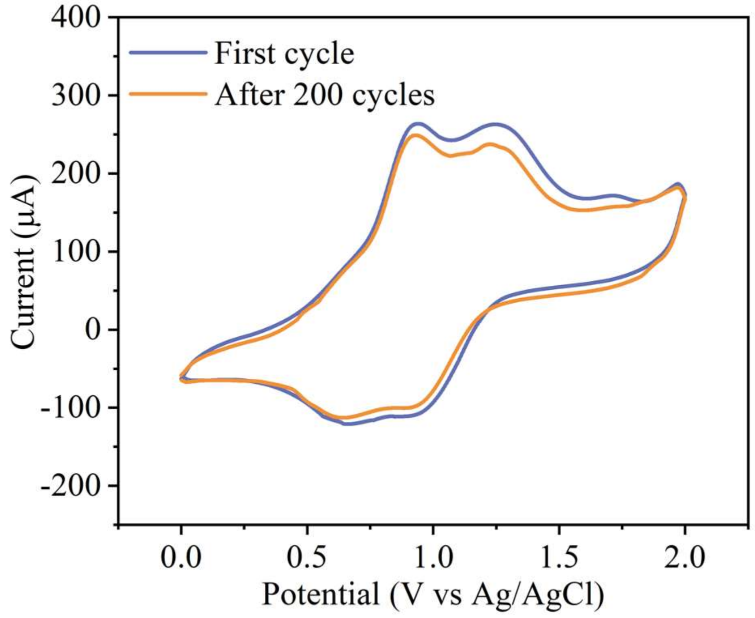

Supplementary Fig. 13. Cyclic voltammogram scans of the **EC-COF-1** film.

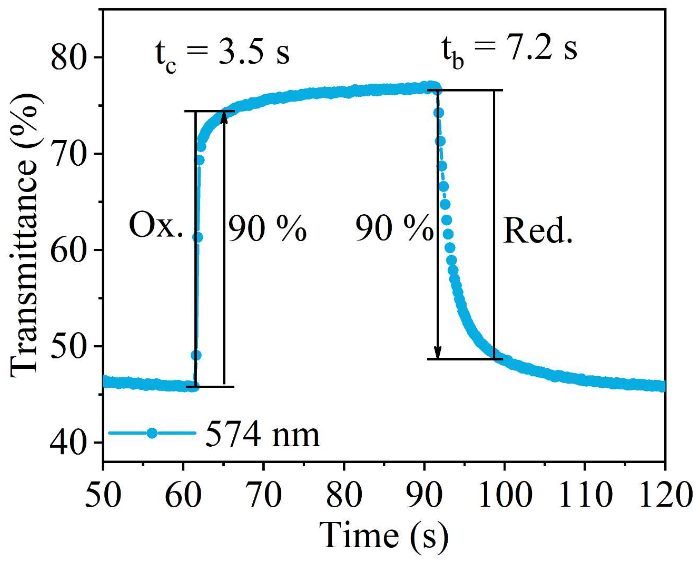

Supplementary Fig. 14. Optical transmittance changes of films monitored at 574 nm. Colouring time (t_c) and bleaching time (t_b) of **EC-COF-1** film electrodes was calculated as 7.2 and 3.5s.

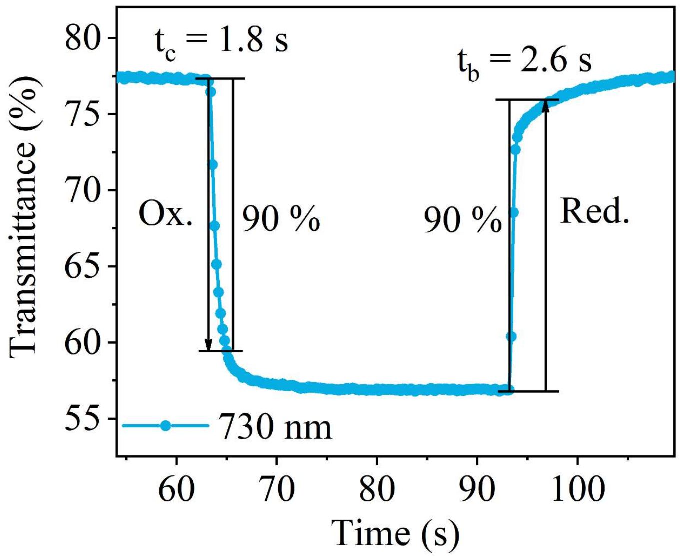

Supplementary Fig. 15. Optical transmittance changes of a film monitored at 730 nm. Colouring time (t_c) and bleaching time (t_b) of **EC-COF-1** film electrodes was calculated as 1.8 and 2.6 s.

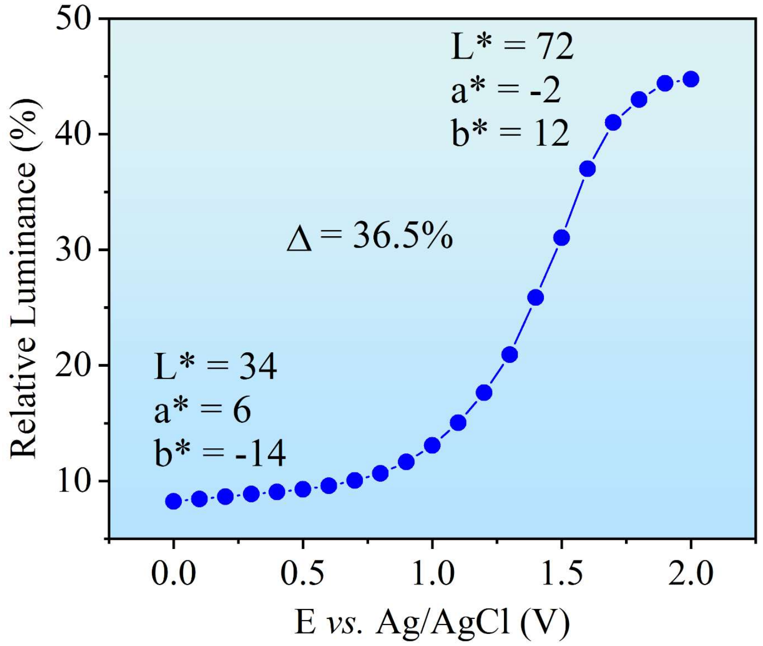

Supplementary Fig. 16. Relative luminance (%) as a function of applied potential for **EC-COF-1**. For colour matching, L*a*b* values (in the sense of the CIE 1976 L*a*b* colour model) of fully neutral and oxidised states are reported.

Supplementary References

1. Lu, C. C. & Su, S. K. Gelation of a highly fluorescent urea-containing triarylamine derivative: N, N, N', N'-tetrakis (*p*-octadecylureido-phenyl)-*p*-phenylenediamine in organic solvents. *Supramol. Chem.* **21**, 547–554 (2009).
2. Tamilavan, V. *et al.* A novel donor-acceptor-acceptor-acceptor polymer containing benzodithiophene and benzimidazole-benzothiadiazole-benzimidazole for PSCs. *Korean Chem. Soc.* **35**, 1098−1104 (2014).
3. Kresse, G. & Furthmüller, J. Efficient iterative schemes for *ab initio* total-energy calculations using a plane-wave basis set. *Phys. Rev. B: Condens. Matter Mater. Phys.* **54**, 11169−11186 (1996).
4. Blöchl, P. E. Projector augmented-wave method. *Phys. Rev. B: Condens. Matter Mater. Phys.* **50**, 17953−17979 (1994).
5. Perdew, J. P., Burke, K. & Ernzerhof, M. Generalized gradient approximation made simple. *Phys. Rev. Lett.* **77**, 3865 (1996).
6. Grimme, S., Antony, J., Ehrlich, S. & Krieg, H. A consistent and accurate ab initio parametrization of density functional dispersion correction (DFT-D) for the 94 elements H-Pu. *J. Chem. Phys*. **132**, 154104 (2010).
7. Heyd, J., Scuseria, G. E. & Ernzerhof, M. Hybrid functionals based on a screened Coulomb potential. *J. Chem. Phys.* **118**, 8207−8215 (2003).
8. Gaupp, C. L., Welsh, D. M., Rauh, R. D. & Reynolds, J. R. Composite coloration efficiency measurements of electrochromic polymers based on 3, 4-alkylenedioxythiophenes. *Chem. Mater*. **14**, 3964–3970 (2002).
9. Reeves, B. D. et al. Spray coatable electrochromic dioxythiophene polymers with high coloration efficiencies. *Macromolecules* **37**, 7559–7569 (2004).
